# Differential responses from the left postcentral gyrus, right middle frontal gyrus, and precuneus to meal ingestion in patients with functional dyspepsia

**DOI:** 10.3389/fpsyt.2023.1184797

**Published:** 2023-05-19

**Authors:** Yiping Chen, Risheng Yu, Joseph F. X. DeSouza, Yuze Shen, Hanyun Zhang, Chunpeng Zhu, Peiyu Huang, Caihua Wang

**Affiliations:** ^1^Department of Psychiatry, Zhejiang University School of Medicine Second Affiliated Hospital, Hangzhou, Zhejiang, China; ^2^Department of Radiology, Zhejiang University School of Medicine Second Affiliated Hospital, Hangzhou, Zhejiang, China; ^3^Department of Psychology and Biology, Neuroscience Graduate Diploma Program and Graduate Program in Interdisciplinary Studies, Multisensory Neuroscience Laboratory, Centre for Vision Research, York University, Toronto, ON, Canada; ^4^VISTA and Canadian Action and Perception Network (CAPnet), Toronto, ON, Canada; ^5^Department of Psychiatry, First People's Hospital, Hangzhou, China; ^6^Department of Gastroenterology, Zhejiang University School of Medicine Second Affiliated Hospital, Hangzhou, China

**Keywords:** functional dyspepsia, functional connectivity, meal ingestion, left postcentral gyrus, right precuneus, right middle frontal gyrus, anterior cingulate cortex, right inferior frontal gyrus

## Abstract

**Background:**

Functional dyspepsia (FD) is most often a meal-induced syndrome. Studies using resting-state functional magnetic resonance imaging (rs-fMRI) reported abnormal connectivity in areas related to pain processing in FD. However, only a few studies have attempted to determine how meal ingestion affects the brain's working patterns. Through rs-fMRI, this study observed how meal ingestion affected brain regions related to visceral hypersensitivity and emotional response networks in FD patients.

**Methods:**

A total of 30 FD patients and 32 healthy controls (HC) were enrolled and underwent clinical investigations. Rs-fMRI was performed twice after a 4-h fast and 50 min after a meal. The mean functional connectivity strength (FCS) values were extracted from brain regions with significant differences to show the trend of changes related to meal ingestion after FCS analyses.

**Results:**

Depression, anxiety, sleep disturbances, and weight loss were more common in FD patients (*P* ≤ 0.001). Compared with HCs (corrected cluster *P*-value < 0.05), FD patients had significantly higher FCS in the right middle frontal gyrus before meals and higher meal-induced FCS in the left postcentral gyrus. HCs had greater meal-induced activation in the right precuneus and anterior cingulate cortex. FD patients had a decreasing trend in the right inferior frontal gyrus compared to the increasing trend in HCs. We only found anxiety to be negatively correlated with FCS in the right inferior frontal gyrus in FD (*r* = −0.459, *p* = 0.048, uncorrected).

**Conclusions:**

In this study, we discovered that FD patients have different perceptual and emotional responses to food intake in defined brain areas, providing promising impetus for understanding pathogenic brain mechanisms in FD.

## Introduction

Functional dyspepsia (FD) is characterized by persistent or recurrent upper abdominal pain or discomfort (1). According to the Rome III criteria for FD, the global prevalence of FD in the community ranges from 5 to 11% (2). Substantial evidence indicates that FD can affect life quality, reduce work attendance and productivity (3), and lead to high financial costs (4). Despite its high worldwide prevalence and dramatic impact on patients, healthcare organizations, and society, the pathophysiology of this disorder remains unclear. It has been suggested that brain–gut axis dysfunction may be one of the pathological mechanisms of FD (5). Therefore, understanding the mechanism of neurological dysfunction in FD may be significant for informing early prognosis and further treatment.

Recent brain imaging studies on FD investigated the central processing of visceral sensation. The results of these studies show not only functional abnormalities in the somatosensory cortex (SI and SII), prefrontal cortex (PFC), thalamus, anterior cingulate cortex (ACC), insula, and amygdala (6–11) but also structural abnormalities in the PFC, ACC, posterior cingulate cortex (PCC), superior parietal cortex (SPC), insula (12, 13). The somatosensory cortex and thalamus are related to the primary processing of visceral senses, while the insula, PFC, and ACC are often related to the advanced integration of sensory-motor, cognitive, and emotional responses (14, 15). A growing body of evidence confirms that PFC dysfunctions cause impairments in emotional memory circuits, and emotional stimuli might trigger increased attentional processing (16, 17) and abnormal functional connectivity of a specific network of brain regions related to many mental diseases (18, 19). In patients with irritable bowel syndrome (IBS), the effects of the limbic region on pain hypersensitivity and emotional response have been fully studied and discussed (20, 21). These findings suggest dysfunctional abnormalities in some brain regions related to homeostatic afferent processing, cognitive regulation, and emotional response in FD.

In previous studies, brain activations related to visceral hypersensitivity in FD were studied using intragastric nutrient infusion or balloon distension to simulate meal ingestion (6, 7, 22, 23). However, it is invasive and does not represent normal meal ingestion. Although FD is often a meal-induced syndrome (24), normal-sized meals often cause discomfort in FD. However, compared with IBS patients, there are relatively few neuroimaging studies during or after food ingestion, and few studies have attempted to determine how food ingestion affects the working brain patterns in FD patients. The mechanisms of intolerance to normal-size meals in FD are still unclear. Therefore, different tests, such as real food intake, are required. The real-meal intervention imaging study could improve our understanding of FD.

In this study, we planned to use resting-state functional magnetic resonance imaging (rs-fMRI) to study brain activity in FD patients before and after meal ingestion. We identified abnormal FCS seed regions through functional connectivity strength (FCS) analysis. We hypothesized that FD patients would exhibit abnormal FCS in brain regions related to visceral hypersensitivity and emotional response networks.

## Materials and methods

### Study protocol

Bisschops et al. systematically investigated the time course of symptoms in relation to meal ingestion in FD patients and found that symptom peaks occurred 40–75 min after a meal (25). This study was designed so that all subjects could be subjected to clinical investigations and psychiatric assessments, and the brain images were collected by RS-fMRI two times to study the responses to a test meal. The subjects were instructed to have their usual breakfast after an overnight fast. The first resting scan was performed 4 h after breakfast. Then, the subjects were required to have a test meal in a quiet room. The postprandial scan was performed 50 min after the ingestion of the test meal.

### Participants

A total of 30 FD patients and 32 healthy controls (HC) were enrolled in our study. All enrolled FD patients were diagnosed by an experienced gastroenterology physician and met Rome III symptom criteria for postprandial distress syndrome (26). We excluded subjects with organic gastrointestinal diseases through clinical investigations. Moreover, we excluded subjects with a history of severe physical and psychiatric diseases; women in pregnancy or lactation who were addicted to illegal drugs or alcohol and unwilling to stop using them during the study period; and subjects who were unable to correctly express their chief complaint and cooperate with the researcher. As left-handedness may affect brain functional analysis, we only enrolled right-handed subjects. All participants were prohibited from taking medications within 24 h before scanning. Eleven FD patients and nine HC patients were excluded from the analysis due to incomplete clinical data collection. Overall, we analyzed the data from 90 FD patients and 23 HC patients, matched for age [*F*_(1, 40)_ = 1.366; *p* = 0.136] and gender[χ^2^_(2, N = 42)_ = 0.006373; *p* = 0.936]. All participants had stable living conditions, excluding the influence of psychosocial factors. The Medical Research Ethics Committee of the hospital approved the research protocol. All participants signed written informed consent.

### Clinical symptoms questionnaires

All participants completed a questionnaire that included information about their sex, age, education, occupation, body mass index (BMI), marital status, financial background, and eating habits in their daily routines. An additional questionnaire for patients with FD to collect information on their symptoms and basic medical status involved the evaluation of the frequency and level of eight FD symptoms. The sum of the scores for the frequency and level of each of the eight FD symptoms is defined as the dyspepsia symptom score (DSS). The patients were asked to provide self-assessments regarding their feelings since the onset of FD, and the scoring was finalized through a discussion between the doctor and patients. In addition, the most serious symptoms, the causes of symptoms, and the factors that exacerbate them were also included.

### Psychosocial assessments

#### SAS and SDS

The participants' levels of depression and anxiety were assessed through the Self-Rating Depression Scale (SDS) (27) and the Self-Rating Anxiety Scale (SAS) (28). The scales include 20 self-assessment items each to evaluate depression and anxiety. Responses were graded on a 4-point scale, with a higher score indicating more obvious symptoms.

#### Pittsburgh Sleep Quality Index

We used the Pittsburgh Sleep Quality Index scale (PSQI) (29) to evaluate sleep quality, including subjective sleep quality, latency, efficiency, persistence, and whether the patients used sleep drugs.

#### SF-36 quality of life questionnaire

The SF-36 Chinese version (30) was used to evaluate the quality of life, which reflects eight aspects of life quality. All subjects were self-evaluated and given the necessary explanation by doctors.

#### Test meal

The test meal was a bowl of Chinese noodles containing 106 g of noodles, dehydrated beef, and vegetables, including cabbage, carrot, shallot, garlic, and chili, with 300 ml of boiled water. Total caloric intake was 490 kcal, with 54 g of carbohydrates, 25 g of lipids, and 7 g of proteins. Noodles were cooked using a standard method, and the subjects were instructed to consume them.

#### Imaging data acquisition

One structural scan and a sequential resting scan (total duration, 27 min) were performed after a 4-h fast (fasting scan), and one resting scan (duration, 20 min) was performed 50 min after a meal (postprandial scan). The subjects were instructed to urinate before undergoing brain imaging to prevent any visceral sensations caused by bladder expansion. During the procedure, they remained awake and lay still on the scanner table with their heads immobilized, eyes blindfolded, and ears plugged. We performed all scans on a 3.0T GE MR scanner in the Department of Radiology, Second Affiliated Hospital, Zhejiang University School of Medicine. We used a 3-D GRE T1-weighted sequence (TR/TE = 8.2/3.2 ms, flip angle = 12°, FOV = 256 × 256 mm^2^, matrix = 256 × 256, slice thickness = 1 mm; no gap, 192 sagittal slices) to acquire anatomical images, and used an echo planar imaging sequence (TR = 2,000 ms, TE = 30 ms, flip angle = 90°, FOV = 240 × 240 mm^2^, matrix = 64 × 64, slice thickness = 3 mm; slice gap = 1 mm; 36 interleaved axial slices) to acquire blood oxygenation level-dependent (BOLD) images.

#### fMRI preprocessing

Resting scan images were preprocessed using a toolbox for Data Processing and Analysis for Brain Imaging (DPABI, http://rfmri.org/dpabi) (31). We removed the first 10 time points for scanner stabilization and preprocessed the remaining images using statistical parametric mapping software, which included co-registration and segmentation of the functional image with the T1-weighted images. These images were then warped into the anatomical Montreal Neurological Institute (MNI) template and transformed into the standard MNI space at a 3 mm isotropic resolution. We used a 6 mm Gaussian kernel to smooth the images, removed linear trends, and performed bandpass filtering (0.01–0.1 Hz), using DARTEL to normalize anatomical images into standard space.

During preprocessing, we excluded subjects with head motion with >2 mm of translation or 2° of rotation to reduce the influence of head motion on FCS calculation. We used frame-wise displacement (32) to evaluate the sum of the absolute values of the six motion parameter derivatives and excluded the time point with a frame-wise displacement >0.5 mm.

#### Functional connectivity analysis

The FCS was calculated using the DPABI tool, which involved the following steps: (1) calculation of Pearson correlations between each voxel and other voxels in the gray matter; (2) retention of all voxels with a correlation factor exceeding the threshold of *r* = 0.25 (33) as functional connections; and (3) summation of all functional connection values for each voxel. The resulting connectivity maps were then subjected to spatial smoothing (full width at half maximum = 6 mm) (34).

First, the generalized linear model was used to perform independent sample *t*-tests to compare and analyze the differences in fasting and postprandial between the FD patients and HC, with age and gender introduced as covariates. Second, we performed paired *t*-tests to observe the differences between fasting and postprandial states in each group, reflecting the effect of ingestion on brain activity. Third, the interaction analysis model was used to observe the interaction between ingestion and groups. We used AlphaSim correction (Alphasim; https://afni.nimh.nih.gov/pub/dist/doc/manual/AlphaSim.pdf) for multiple comparison correction and set the statistic threshold at a corrected cluster *P*-value of < 0.05.

After a brain analysis, a *post-hoc* analysis of the different brain regions was performed. We selected brain regions with significant differences to create spherical regions of interest (ROIs). These ROIs were then used as seed regions for seed-based FC analysis. FCS averages were extracted from these regions to show the trend of changes.

#### Demographic data statistical analysis

We used SPSS 22.0 to analyze demographic and clinical data and performed a one-way analysis of variance to compare ages between FD patients and the HC group. We performed a Chi-square test to compare the sex differences and used a two-sample *t*-test to compare the scale scores between the two groups. The categorical data were described by the frequency (percentile, %), and the numerical data were described with the mean (standard deviation, SD).

## Results

### Demographic and clinical data

Table 1 lists the demographics and clinical data of FD patients and HC patients. We excluded 9 HC and 11 FD patients from the data analysis for incomplete data collection (*n* = 9) and excess head motion (*n* = 11). Thus, the data from 23 HC and 19 FD patients were analyzed. No significant differences were found in sex and age between FD patients and HC patients (*P* > 0.05). BMI scores were significantly lower in FD patients than in HC patients (20.24 ± 3.04 vs. 22.93 ± 2.78; *P* = 0.001). FD patients had statistically significantly higher anxiety (49.85 ± 8.32 vs. 32.75 ± 7.68; *P* < 0.001), depression (52.59 ± 7.73 vs. 40.65 ± 10.51; *P* < 0.001), and PSQI scores (8.95 ± 3.50 vs. 3.76 ± 1.90; *P* < 0.001) than HC patients.

**Table 1:** Demographic and clinical data of the FD patients and HCs.

**Protocols**	**FD (*n* = 19)**	**HC (*n* = 23)**	***P*-value**
Gender (M/F)	13/6	16/7	>0.1
Age (years)	35.42 ± 10.55	34.22 ± 9.19	>0.1
BMI	20.24 ± 3.04	22.93 ± 2.78	0.001
SDS	52.59 ± 7.73	40.65 ± 10.51	< 0.001
SAS	49.85 ± 8.32	32.75 ± 7.68	< 0.001
PSQI	8.95 ± 3.50	3.76 ± 1.90	< 0.001

Nineteen FD patients and 23 HC patients out of 62 subjects provided data suitable for analysis. Values are expressed as mean ± SD.

After a brain map analysis and *post-hoc* analysis of the different brain regions, we found five brain regions with significant FCS differences between FD and HC before and after meal ingestion: the left postcentral gyrus (PostCG), the right precuneus (PCu), the right middle frontal gyrus (MFG), the anterior cingulate cortex (ACC), and the right inferior frontal gyrus (IFG). Regions of interest (ROI) were defined from the significant cluster, and the mean FCS values were extracted from them to show the trend of changes. The size and peak coordinates of these areas are shown in Table 2.

**Table 2:** Brain regions represent FCS differences between FD patients and HCs.

**Contrast**	**Brain regions**	**MNI coordinates (x, y, z) (mm)**	**Numbers of voxels**	**Peak*t*-value^a^**
FD_post vs. FD_pre	PostCG (L)	(−51, −21, 51)	40	4.536
FD_pre vs. HC_pre	MFG (R)	(30, 18, 57)	81	4.528
FD_post vs. HC_post	ACC	(−3, 30, 18)	33	−4.073
HC_post vs. HC_pre	PCu (R)	(6, −72, 45)	50	4.675
Groups^*^ food interaction	IFG (R)	(39, 30, −12)	37	−4.325

Five brain regions showed significant FCS differences between FD and HC before and after meal ingestion. HC_pre, HCs in the pre-meal state; HC_post, HCs in the post-meal state; FD_pre, FD patients in the pre-meal state; FD_post, FD patients in the post-meal state; Groups^*^, FD (post - pre) vs. HC (post – pre); L, left; R, right.

^a^Negative value represents lower FCS; a positive value represents higher FCS.

### FCS in the left PostCG

**Figure 1:**
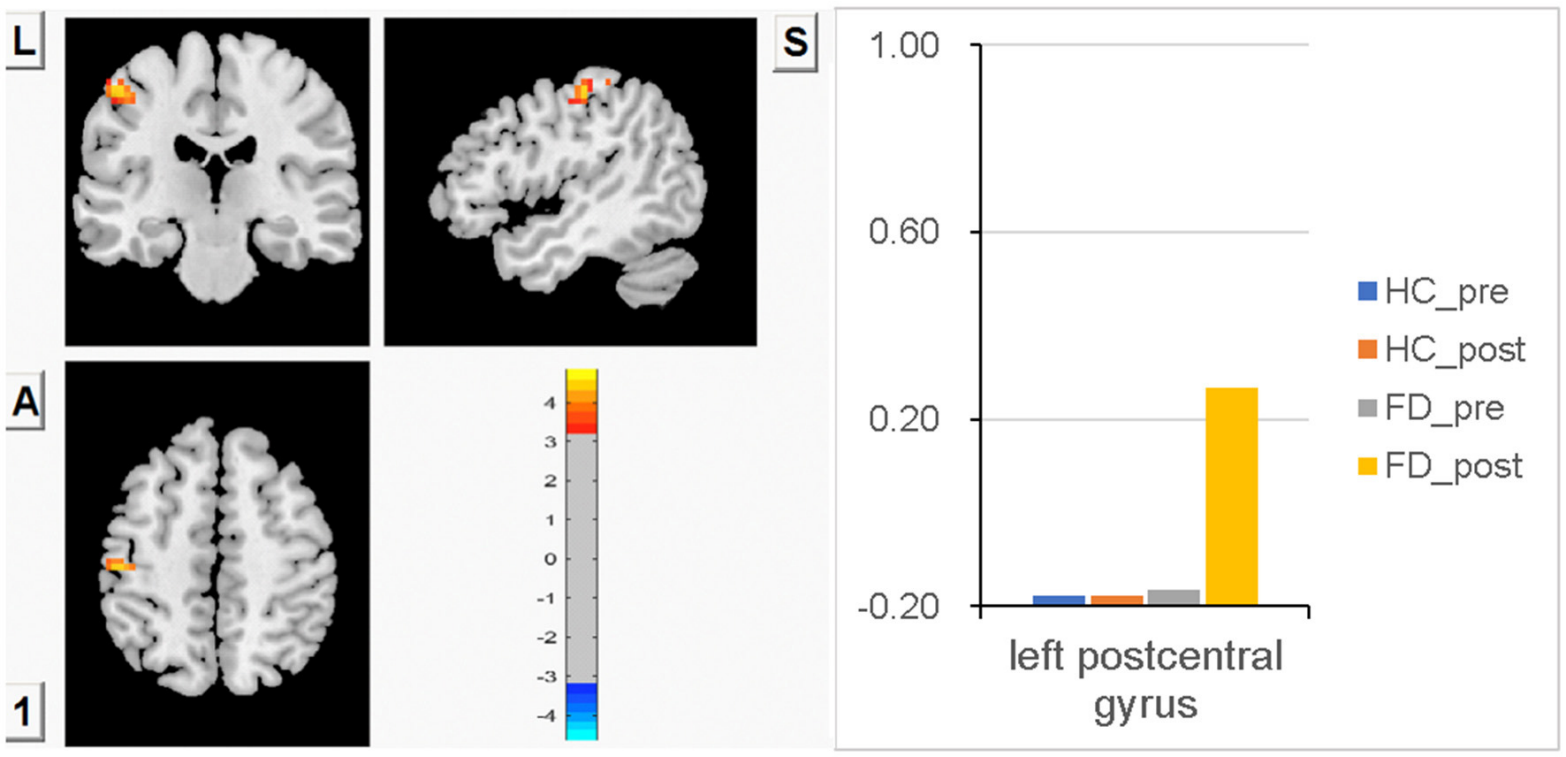
There is no significant difference in FCS in the left PostCG between FD patients and HC before a meal, and meal-induced FCS increases are significant in FD patients, while there is no significant difference between fasting and postprandial in HC.

No significant difference in FCS in the left PostCG was observed between FD patients and HC before a meal, and significant meal-induced FCS increases were observed in FD patients, which were significantly higher than fasting, but no significant difference was observed between fasting and postprandial in HC. FCS differences of the left PostCG between fasting and postprandial states in the HC and FD groups are shown in Figure 1. Color bars show the trend of FCS changes between groups before and after meal ingestion. The orange-red area in Figure 1 showed greater FCS increases in the left PostCG, which indicates the impact of meal ingestion in FD patients. The size and peak coordinates of the left PostCG in FD patients are shown in Table 2.

### FCS in the right MFG

**Figure 2:**
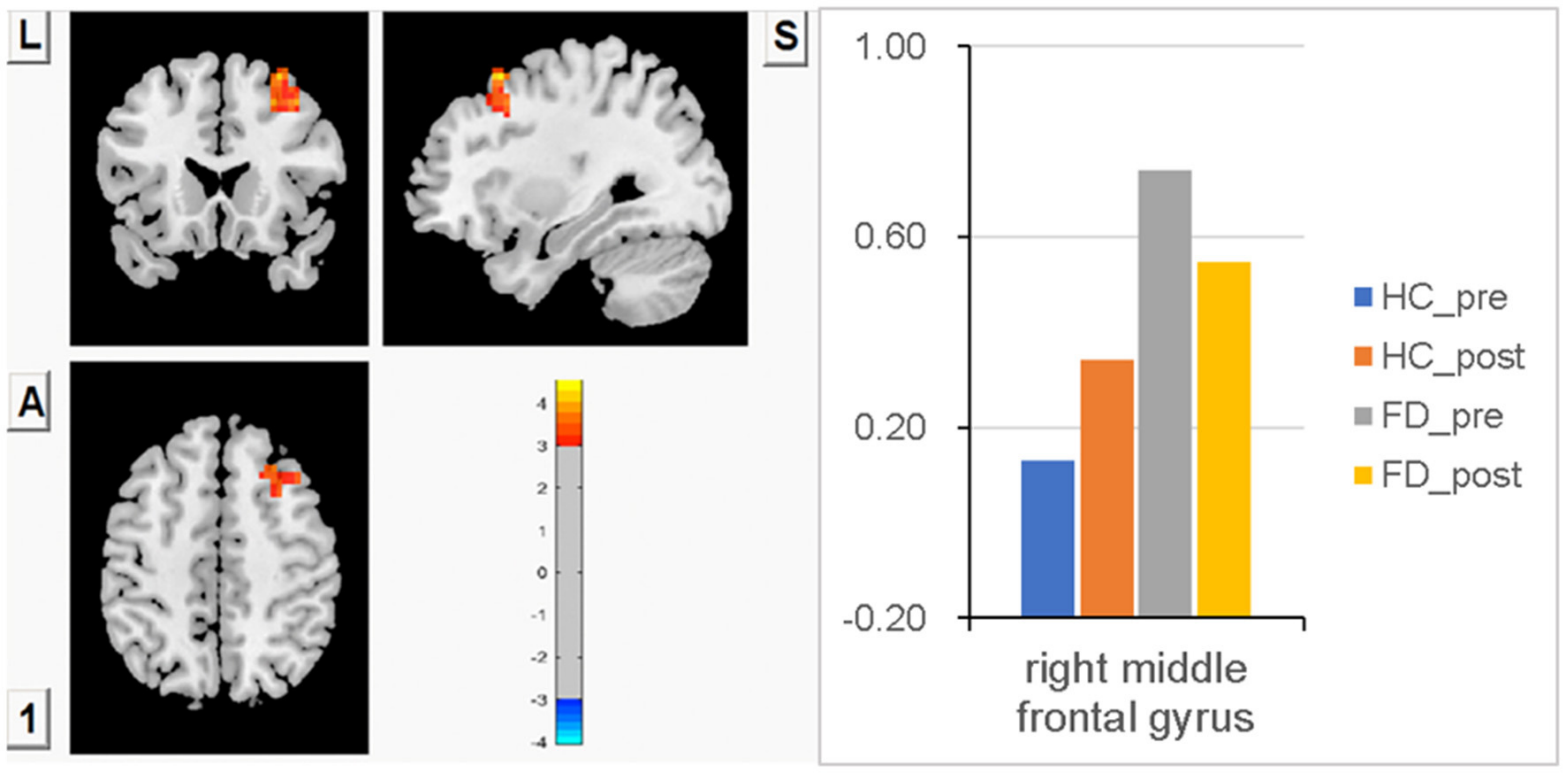
Significantly higher FCS in the right MFG before a meal, meal-induced FCS decreases in FD, meal-induced FCS increases in HC, but no significant difference within the group both in FD and HC, and no significant FCS difference in the postprandial state between HC and FD patients.

Compared with HC, significantly higher FCS in the right MFG was observed in FD patients before a meal. After meal ingestion, meal-induced FCS decreases in the right MFG were observed in FD. In contrast, meal-induced FCS increases in the right MFG were observed in HC, but no significant difference within the group was observed in FD patients or HCs. No significant FCS difference in this area was observed between HC and FD patients in a postprandial state. FCS differences of the right MFG between fasting and postprandial state in the HC and FD groups are shown in Figure 2. The orange-red area showed greater FCS increases in the right MFG. Color bars show the trend of FCS changes in the right MFG between groups before and after meal ingestion. The size and peak coordinates of the right MFG in FD patients are shown in Table 2.

### FCS in ACC

**Figure 3:**
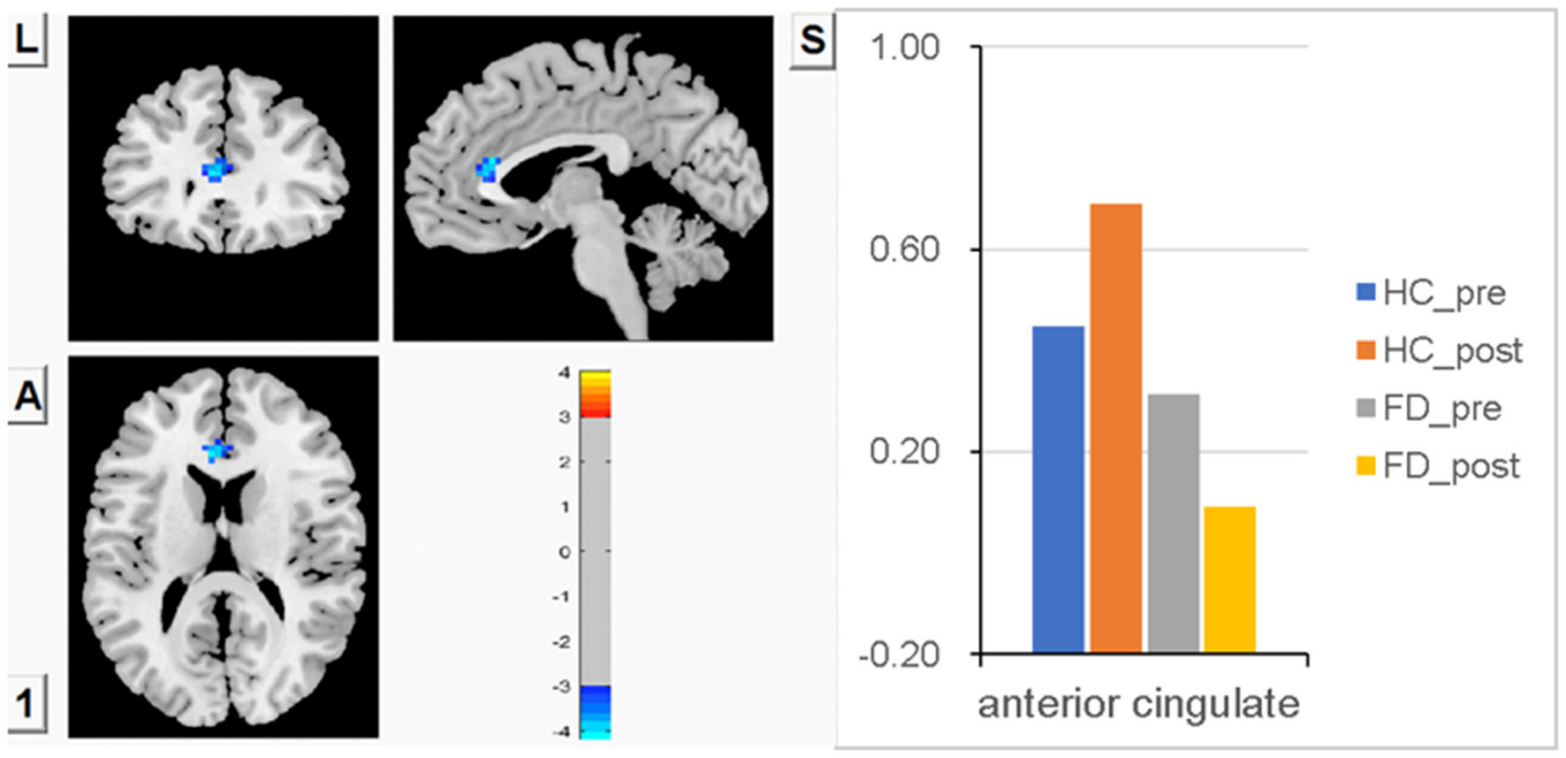
There is no significant difference in FCS in ACC between the two groups before a meal, no significant difference in meal-induced FCS increases within the HC group, and no significant difference in meal-induced FCS decreases within the FD group, but significantly higher meal-induced FCS increases in HC compared with FD patients.

No significant difference in FCS in ACC was observed between FD patients and HC patients before a meal. Meal-induced FCS increases were observed in HC, with no significant difference within the group. In FD patients, meal-induced FCS decreases were also observed, with no significant difference within the group. But after meal ingestion, significantly increased FCS in ACC was observed in HC compared with FD patients. FCS differences in ACC between fasting and postprandial states in the HC and FD patient groups are shown in Figure 3. The blue area showed greater FCS decreases in ACC in FD patients. Color bars show the trend of FCS changes between groups before and after meal ingestion. The size and peak coordinates of ACC are shown in Table 2.

### FCS in the right precuneus

**Figure 4:**
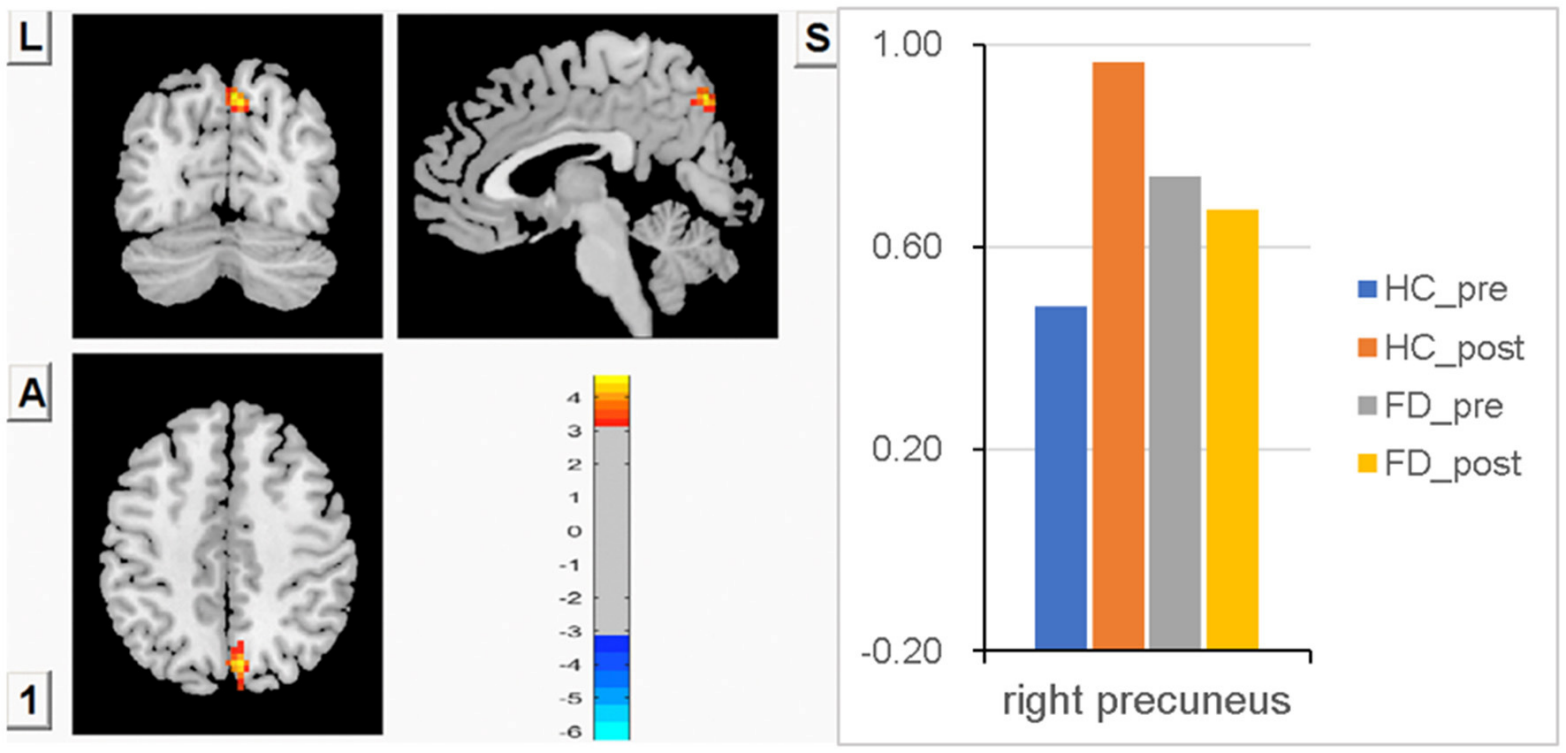
Significant meal-induced FCS increases in the right precuneus in HC, while no significant meal-induced difference in FD patients.

Compared with FD patients, lower FCS in the right precuneus was observed in HC before a meal, though there was no statistically significant difference between groups. After meal ingestion, significant meal-induced FCS increases in the right precuneus were observed in HC, which were significantly higher than in fasting, while no significant difference in the right precuneus was observed between fasting and postprandial in FD patients. FCS differences in the right precuneus between fasting and postprandial states in HC and FD patients are shown in Figure 4. The orange-red area showed greater FCS increases in the right precuneus, which indicates the impact of meal ingestion on HC. Color bars show the trend of FCS changes in the right precuneus between groups before and after meal ingestion. The size and peak coordinates of the right precuneus in HC and FD patients are shown in Table 2.

### Analysis of the interaction effect of FCS changes in the right IFG

**Figure 5:**
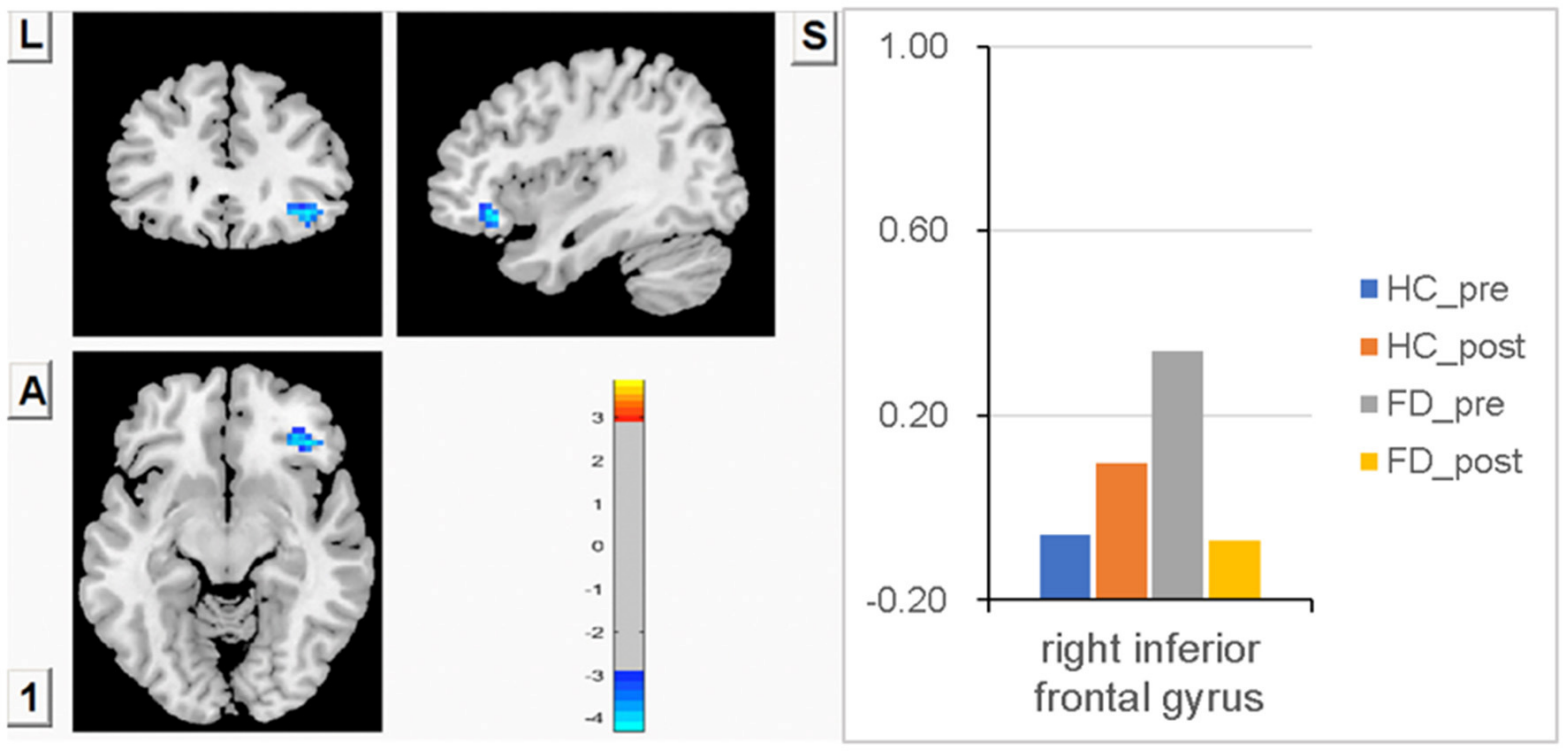
Higher FCS in the right IFG before a meal in FD, but no statistically significant difference between groups. There was a decreasing trend of meal-induced FCS in FD and an increasing trend in HC, and the changes in meal-induced FCS in the right IFG had statistical significance between groups.

Higher FCS in the right IFG before a meal was observed in FD compared with HC, but there was no statistically significant difference between groups. After meal ingestion, FCS in the right IFG observed a decreasing trend in FD, while FCS in the HC had an increasing trend, but no statistically significant changes were found within each group. The changes in FCS in the right IFG were found to be statistically significant between FD patients and HC patients after meal ingestion. FCS differences of the right IFG between fasting and postprandial state in HC and FD patients are shown in Figure 5. The blue area showed greater FCS decreases in the right IFG. Color bars show the trend of FCS changes in the right IFG between groups before and after meal ingestion. The size and peak coordinates of the area are shown in Table 2.

### Relationship between clinical indices and changes in FCS

**Figure 6:**
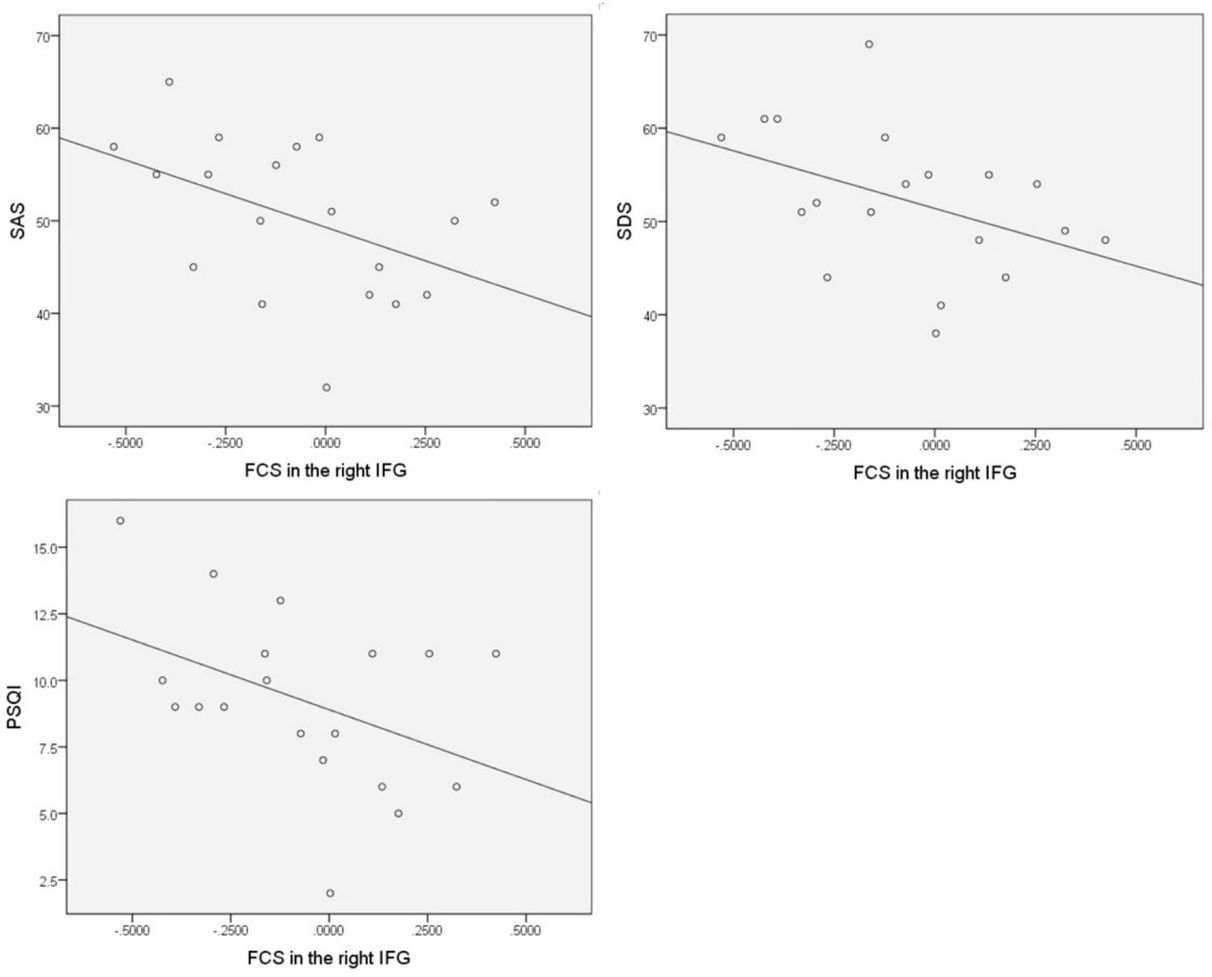
SAS scores of FD patients negatively correlated with FCS in the right IFG, and FCS in the right IFG showed a negative correlation trend with scores of SDS but with no statistically significant correlation after the correction by age and gender.

All brain regions with significant FCS differences were examined for relationships with the disease duration of FD patients, scores of FD symptoms, PSQI, SAS, and SDS, and multiple regression analyses revealed that SAS scores of FD patients were negatively correlated with FCS in the right IFG (*r* = −0.459, *p* = 0.048), and FCS in the right IFG showed a negative correlation trend with scores of SDS (*r* = −0.428, *p* = 0.068), PSQI (*r* = −0.423, *p* = 0.071) (Figure 6). However, no statistically significant correlation was observed after the correction by age and gender, and we did not find FCS in other brain regions that showed significant correlations with the disease duration, scores of FD symptoms, PSQI, SAS, and SDS (*p* > 0.05).

This study found depression, anxiety, sleep disturbances, and weight loss were more common in FD patients. Compared with controls, FD patients had significantly higher FCS in the right MFG before meals. After meal ingestion, we observed higher meal-induced FCS in the left postCG in FD patients and greater activation in the right precuneus and ACC in controls. We found a decreasing trend in the right IFG in FD patients compared to the increasing trend in controls. In accounting for psychological factors, we only found that anxiety was negatively correlated with FCS in the right IFG in FD patients, but no statistical differences were observed after the correction by age and gender.

## Discussion

Healthy people can ingest normal-sized foods without any discomfort. Previous positron emission tomography (PET) studies have reported that “pain neuromatrix” regions were progressively deactivated during nutrient infusion in the HC group (35). According to Huynh Giao Ly's nutrient-induced distension study in HC, progressive activation was found in the MFG, midbrain, thalamus, MCC, superior temporal gyrus, and cerebellum with increasing gastric distension level, while progressive deactivation was found in regions associated with the “pain neuromatrix,” including the secondary somatosensory cortex, ventromedial and ventrolateral prefrontal cortex, and insula (10). Compared to the HC group, the activation of the somatosensory cortex, PFC, thalamus, and insula in FD patients was consistently greater, while brain activities of the orbitofrontal cortex (OFC) and ACC were not consistent (9, 10, 36–42). During distension research in hypersensitive FD, compared to HC, activation was found in the ventrolateral prefrontal cortex and somatosensory cortex but not in pregenual ACC (6).

In this study, we did not observe a significant difference in FCS in the somatosensory cortex between FD patients and HC during the fasting state, but we observed group differences in the left PostCG during ingestion. Significant meal-induced FCS increases were observed in FD patients during ingestion, which were significantly higher than the fasting state, while no significant difference was observed between fasting and postprandial in HC. This finding may suggest that this region is sensitized or receives increased ascending input during ingestion in FD. Since FD is a chronic disease, these different brain activities in the process of visceral afferent signal transmission indicate that FD patients may have enhanced visceral hypersensitivity, and persistent afferent visceral sensory input to the somatosensory cortex may lead to abnormal central regulation (43, 44). Our findings indicated that individuals with FD have an enhanced visceral perception, as evidenced by greater activity in the somatosensory cortex following meal stimulation. This heightened perception may contribute to discomfort reactions in response to normal-sized foods and supports the hypothesis of cortical sensitization in FD.

The frontal cortex is responsible for the integration of peripheral information, cognitive modulation of pain (medial, dorsolateral), and emotional response to pain sensation (medial, ventrolateral) (45). The brain regions that process visceral sensations are closely related to the emotional regulation regions (46). It is well known that functional gastrointestinal diseases are often accompanied by depression, anxiety, and somatization (47). Many researchers have found that depressed patients have functional changes in dlPFC (48, 49), MFG (50), and precuneus (51–53), which may lead to emotionally related behavioral dysfunction. In fMRI studies of MDD, it has often been observed that with higher functional activity in the DLPFC (54), MFG regulates the intensity of responses to emotional events. Abnormal MFG function may lead to emotional instability (22). Compared to the previous study in the HC group, progressive MFG activation with increasing distension levels has been reported during nutrient infusion (34). In this study, we found that the brain activation of PD patients is stronger than that of the HC group in the lateral prefrontal cortex; group differences in brain responses during the fasting state were observed in the right MFG; and significantly higher FCS in FD patients suggests that this region is sensitized without any peripheral stimulus, which may indicate that emotional arousal is stronger in FD patients. We believed that the abnormal function of the MFG may cause FD patients to be more susceptible to emotional issues. We also observed a non-significantly higher FCS in the right IFG during fasting. We found the deactivation pattern of FCS in the right IFG is similar to MFG in response to a meal stimulus. Both right MFG and IFG postprandial FCS showed a decreasing trend in FD patients. We speculated that this could be due to the ceiling effect since MFG-related brain regions have been highly activated and instead tend to attenuate in FD patients under the peripheral stimulus. It could be related to the overlapping effects of visceral sensory afferents, cognitive processing, and pain regulation. In this study, we found that FCS in the right MFG showed an increasing trend during meal ingestion in HC, but there is no statistical significance. We speculated that it might be due to the small sample size of this study.

ACC is involved in visceral sensation and motivation (55). In this study, we did not find a significant difference in FCS in ACC between FD patients and HC patients during the fasting state, and we observed a non-significantly higher meal-induced FCS within the HC group while a non-significantly lower meal-induced FCS within the FC group. However, after meal ingestion, significantly higher FCS in ACC was observed in HC compared with FD patients. We speculated that the failure to activate ACC during ingestion in FD patients may result in the deactivation of the dorsal pons, which may represent a failure in emotionally driven pain modulation and may explain the postprandial discomfort in FD patients.

Precuneus, the posterior core of the default mode network, is related to visual-spatial images, episodic memory retrieval, and future episodic thought (56). Previous studies have reported that eudaemonic wellbeing was positively correlated with the intrinsic functional connectivity of precuneus and VMPFC and confirmed the role of precuneus in a person's enjoyment of eudaemonic wellbeing (57–60). In this study, we made an interesting finding that, despite minor differences between FD patients and HC patients, the process of ingestion resulted in prolonged sensations of fullness and satiation, and significant meal-induced increases in FCS in the right precuneus were observed in the HC group, whereas no significant change in FD was observed. We speculated that it may be related to meal-induced satiety or wellbeing in the HC group. This finding can explain why healthy people are able to enjoy the pleasure of eating. But for FD patients, eating not only prevents them from enjoying food but also increases pain.

Psychological distress, including anxiety, depression, and disordered sleep, is more common in FD patients than in HC patients (21). This study considered depression, anxiety, and sleep disorders as potential factors that may influence brain activity. All brain regions with significant FCS differences were examined for relationships with the disease duration of FD patients, scores of FD symptoms, PSQI, SAS, and SDS. Multiple regression analyses revealed that SAS scores of FD patients were negatively correlated with FCS in the right IFG, and FCS in the right IFG showed a negative correlation trend with scores of SDS and PSQI. However, no statistically significant correlation was observed after the correction by age and gender, and we did not find FCS in other brain regions that showed significant correlations with the disease duration, scores of FD symptoms, PSQI, SAS, and SDS.

The orbitofrontal cortex extends to the laterally adjacent IFG. In patients with depression, FCS changes between the orbitofrontal cortex, IFG, and other brain systems have already been implicated (61–64). However, we did not find statistically significant correlations between psychological factors and IFG in FD after the correction by age and gender. We speculate that this might be due to the small sample size of this study and the mild degree of depression and anxiety in FD, which did not show clear brain changes related to depression.

## Conclusion

Our findings demonstrate different brain responses to fasting and meal ingestion between FD patients and HC patients. We found increased FCS in emotional arousal cortices during fasting and meal-induced FCS in networks processing interoceptive information. Moreover, we found that it decreased meal-induced FCS in areas related to cortico-limbic pain modulatory systems and the extended reward network. These findings indicate that FD patients have different brain responses to food ingestion, suggesting abnormal brain activities in visceral sensory and emotional responses, and provide new neuroscientific evidence for the hypothesis of cortical sensitization in FD patients and more potential biomarkers for treatment.

## Limitations and future directions

This study has some limitations, such as a small sample size, preferences for test meals, and changes in psychological factors caused by some life events that may affect brain activity. There may be some interactions between the severity of FD symptoms, the disease duration of FD, personality traits, eating habits, psychological factors, and brain activity. Owing to limited patient resources, we were not able to explore the impact of these interactions in the current study. In future research, we can expand the sample size, provide more abundant diet choices, analyze personality characteristics, use more diverse neuroimaging tasks, and use different interventions, including antidepressants and non-invasive brain stimulation, to improve our understanding of FD.

## Data availability statement

The raw data supporting the conclusions of this article will be made available by the authors, without undue reservation.

## Ethics statement

The studies involving human participants were reviewed and approved by the Second Affiliated Hospital of Zhejiang University, Medical Research Ethics Committee. The patients/participants provided their written informed consent to participate in this study.

## Author contributions

CW and RY guided the study. YC, YS, HZ, and CZ enrolled the participants and collected data. PH and JD analyzed the data. YC wrote the manuscript. All authors reviewed the manuscript. All authors contributed to the article and approved the submitted version.
